# The delivery of mRNA to colon inflammatory lesions by lipid-nano-particles containing environmentally-sensitive lipid-like materials with oleic acid scaffolds

**DOI:** 10.1016/j.heliyon.2018.e00959

**Published:** 2018-12-03

**Authors:** Hiroki Tanaka, Ayaka Watanabe, Manami Konishi, Yuta Nakai, Hiroki Yoshioka, Tatsuya Ohkawara, Hiroshi Takeda, Hideyoshi Harashima, Hidetaka Akita

**Affiliations:** a Laboratory of DDS Design and Drug Disposition, Graduate School of Pharmaceutical Sciences, Chiba University, 1-8-1, Inohana, Chuo-ku, Chiba-shi, Chiba 260-8675, Japan; b Laboratory for Molecular Design of Pharmaceutics, Faculty of Pharmaceutical Sciences, Hokkaido University, Kita12 Nishi6, Kita-ku, Sapporo City, Hokkaido 060-0812, Japan; c DDS Research Laboratory, DDS Development Division, NOF CORPORATION, 3-3 Chidori-cho, Kawasaki-ku, Kawasaki, Kanagawa 210-0865, Japan; d Laboratory for Pathophysiology and Therapeutics, Faculty of Pharmaceutical Sciences, Hokkaido University, Kita12 Nishi6, Kita-ku, Sapporo City, Hokkaido 060-0812, Japan

**Keywords:** Materials chemistry, Pharmaceutical science

## Abstract

An mRNA gene therapy represents a potentially promising therapeutic for curing inflammatory diseases. The transient nature of the gene expression of mRNA would be expected to be beneficial for avoiding undesired side effects. Since the mRNA is a vulnerable molecule, a development of a carrier that can deliver the mRNA to the cytoplasm has a high priority. We report herein on the development of a system for delivering mRNA to the inflammatory lesion in a dextran sulfate sodium (DSS)-induced colitis model. We modulated molecular structures of an ionizable lipid, an SS-cleavable and pH-activated lipid-like material (ssPalm). Among the fatty acids investigated, oleic acid scaffolds (ssPalmO) appeared to be more biocompatible than either myristic acid or linoleic acid scaffolds with the colitis model. The structural modification of the hydrophilic head groups from linear tertiary amines to piperazine rings (ssPalmO-Paz4-C2) resulted in a more than 10-fold higher increasing in the transgene activity in inflammatory colon. The most notable observation is that the transgene activity in the inflammatory colon is significantly higher than that in liver, the major clearance organ of lipid nanoparticles. Collectively, the ssPalmO-Paz4-C2 represents a promising material for the delivery of an mRNA to inflammatory lesions.

## 1 Introduction

An mRNA therapeutics would be an alternative approach to the conventional gene therapy that use plasmid DNA (pDNA) from the point of view of a treatment for inflammatory diseases. The transient nature of the protein expression of mRNA, generally from several hours to days [1], would be expected to be especially beneficial to the treatment for the inflammatory diseases since a sustained expression of an immune-suppressive or an anti-apoptotic protein is attended by the risk of increased susceptibility to infections or the development of cancer. An mRNA therapy for an asthma model was recently reported [2]. In this research, the pathological over-activation of the Th2 responses were successfully down-regulated by the intratracheal administration of a chemically modified mRNA encoding FoxP3, a characteristic transcription factor produced by regulatory T cells. More recently, the amelioration of a mouse fulminant hepatitis model was also reported [3]. Fas-ligand-induced apoptotic cell death in liver was successfully suppressed by the hydrodynamic injection of nanomicelle-encapsulating mRNA encoding an anti-apoptotic protein Bcl-2. These outcomes clearly indicate the potential feasibility of the use of mRNA therapies for curing these intractable diseases.

**Fig. 1:**
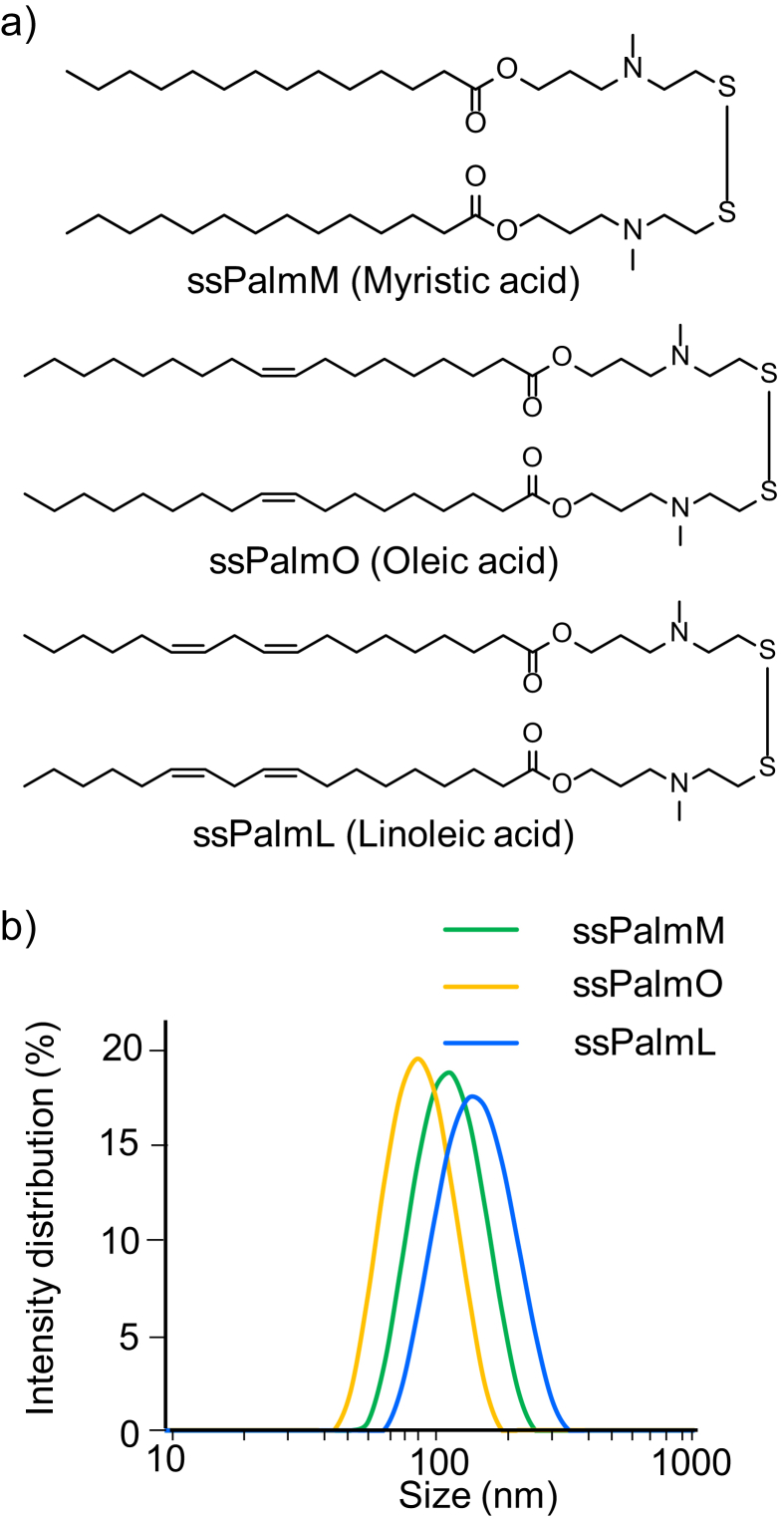
a) Chemical structure of a series of ssPalm (ssPalmM, ssPalmO, and ssPalmL). The ssPalms used in the present study contained myristic acid, oleic acid or linoleic acid as a fatty acid scaffold. The synthesis of the ssPalmO and ssPalmL was described in Supplementary materials. b) Size distribution of the LNP_ssPalms_ prepared by ethanol dilution method. The size distribution was determined by dynamic light scattering. Detailed particle data were summarized on Table 1.

We previously reported that a lipid-nano-particle (LNP) administrated via tail vein spontaneously accumulated in an inflammatory lesion of a dextran sulfate sodium-induced colitis (DSS-colitis) via leaky blood vessels under inflammatory conditions. We demonstrated that an 110 nm sized LNP accumulated in the colon to a higher extent compared to either smaller (54 nm) or larger (180 nm) size particles [4]. We therefore hypothesized that an mRNA carrier with an optimal size (approximately 110 nm) would be a suitable platform for a systemic mRNA delivery for inflammatory diseases. As a material for the mRNA-encapsulating LNP, we have developed a series of ionizable lipids referred to as SS-cleavable and pH-activated lipid-like material (ssPalm, Fig. 1a) [5]. The ssPalm contains double hydrophobic scaffolds for stabilizing the lipid envelope structure and dual sensing motifs that can respond to intracellular environments; ionizable tertiary amines and a cleavable disulfide bonding. Since the ssPalm enables an organization of a neutrally charged LNP (LNP_ssPalm_) at physiological pH, LNP_ssPalm_ can avoid from an undesired formation of large aggregates with erythrocyte and/or platelet in blood stream [6]. On the other hand, once taken up by cells, the ssPalm develops a positive charge via protonation of the amine structures in cellular acidic environments such as endosomal compartment. The positive charge acts as a driving force for endosomal membrane-destabilization and subsequent cytoplasmic delivery of the mRNA. In the cytoplasm, the reductive cleavage of the ssPalm by cellular reducing agent (i.e. glutathione) results in the spontaneous decapsulation of the mRNA, which is expected to be beneficial to the translation process of the mRNA. The objective of this study was to develop an LNP_ssPalm_ that would permit an mRNA to be delivered to inflammatory lesions. We initially selected the hydrophobic scaffolds of the ssPalm from the point of view of biocompatibility with mice under an induced pathological condition (DSS-colitis). We next modified the structure of the tertiary amines to enhance endosomal escape efficiency and subsequent transgene activity in the inflammatory colon.

## 2 Materials and methods

### 2.1 Materials

The ssPalm materials are manufactured by the NOF CORPORATION. ssPalmM (Product # COATSOME^®^ SS-14/3AP-01) was synthesized as described previously [5]. The synthesis of ssPalmO (Product # COATSOME^®^ SS-18/4PE-13), ssPalmO-Paz4-C2 (Product # COATSOME^®^ SS-18/1PZ-23), and ssPalmL (Product # COATSOME^®^ SS-18/4PE-16) was described in the section below. The chemical structures of a series of ssPalm materials used in this study are shown on Fig. 1a. 1,2-dioleoyl-sn-glycero-phosphatidylcholine (DOPC) was purchased from Avanti Polar lipids (Alabaster, AL, USA). 1-(monomethoxy polyethylene glycol 2000)-2,3-distearylglycerol (PEG_2000_-DSG) and 1-(monomethoxy polyethylene glycol 5000)-2,3-distearylglycerol (PEG_5000_-DSG) were purchased from the NOF CORPORATION (Kanagawa, Japan). Dextran sulfate sodium (DSS, M.W. 36,000–50,000) was purchased from MP Biomedicals LLC (Solon, OH, USA).

### 2.2 Synthesis of the ssPalmO, ssPalmO-Paz4-C2, and ssPalmL

#### 2.2.1 General procedures

**Scheme 1:**
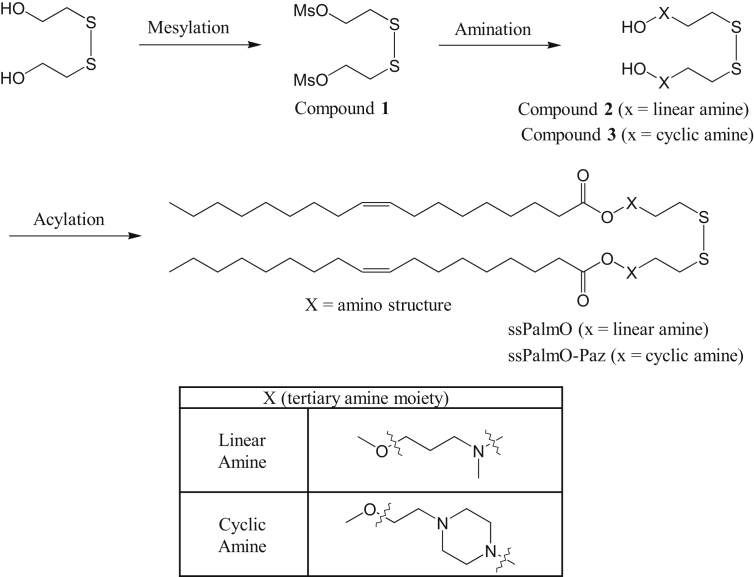
Syntheses of ssPalms. Scheme for the synthesis of the ssPalmO and ssPalmO-Paz4-C2 is shown. ssPalms were synthesized according to the *Synthesis of the ssPalmO, ssPalmO-Paz4-C2, and ssPalmL* section.

All reagents were obtained from commercial sources and were used without further purification. Thin layer chromatography was performed on Merck TLC plates silica gel 60. ^1^H-NMR spectra were recorded on a JEOL ECA600 (1H 600 MHz) spectrometer. Scheme for the synthesis of the ssPalmO and ssPalmO-Paz4-C2 is shown in Scheme 1. Intermediate Compounds **1** and **2** were synthesized as described in a previous report [5]. Compound **1** (1.2 g, 3.9 mmol) was dissolved in acetonitrile (31 mL) at 20–25 °C, and potassium carbonate (1.3 g, 9.7 mmol) was then added. After stirring the mixture at 20–25 °C for 5 minutes, 1-piperazineethanol (5.0 g, 39 mmol) was added and the resulting solution was stirred at 25–35 °C for 13 hours. The potassium carbonate was removed by filtration, and the filtrate was evaporated to give a brown liquid, which was then dissolved in chloroform (25 mL), and the chloroform phase washed with distillated water (3 × 25 mL). The organic layer was dried over magnesium sulfate (0.60 g), the solution was filtered and evaporated to yield compound **3** (1.0 g).

### 2.3 Synthesis of ssPalmO

Compound **2** (0.45 g, 1.5 mmol) and Oleic acid (0.90 g, 3.2 mmol) were dissolved in chloroform (4.5 mL) at 20-25 °C. Thereafter, 4-dimethylamino pyridine (0.074 g, 0.61 mmol) and 1-ethyl-3-(3-dimethylaminopropyl) carbodiimide hydrochloride (0.87 g, 4.6 mmol) were added to the solution. The mixture was then stirred at 30 °C for 3 hours. The resulting mixture was evaporated to give a pale brown liquid. The concentrate was dissolved in hexane (7.5 mL), and acetonitrile (3.5 mL) was added to the solution. The hexane layer was recovered, and evaporated to give the crude ssPalmO as a pale-yellow liquid. The crude material was purified by silica gel column chromatography to yield ssPalmO (0.90 g). ^1^H NMR (600 MHz, CDCl_3_): δ (ppm) = 0.86–0.90 (t, 6H), 1.20–1.35 (m, 40H), 1.58–1.70 (m, 4H), 1.75–1.83 (m, 4H), 1.95–2.05 (m, 8H), 2.24–2.32 (m, 10H), 2.41–2.48 (m, 4H), 2.66–2.70 (m, 4H), 2.78–2.82 (m, 4H), 4.10–4.13 (t, 4H), 5.13–5.38 (m, 4H).

### 2.4 Synthesis of ssPalmO-Paz4-C2

Compound **3** (0.80 g, 2.1 mmol) and Oleic acid (1.2 g, 4.2 mmol) were dissolved in chloroform (8.0 mL) at 20-25 °C. Thereafter, 4-dimethylamino pyridine (0.10 g, 0.85 mmol) and 1-ethyl-3-(3-dimethylaminopropyl) carbodiimide hydrochloride (1.2 g, 6.3 mmol) were added to the solution. The mixture was then stirred at 30 °C for 3 hours. The resulting mixture was evaporated to give a pale brown liquid. The concentrate was dissolved in hexane (12 mL), and acetonitrile (5.0 mL) was added to the solution. The hexane layer was recovered, and evaporated to give the crude ssPalmO-Paz4-C2 as a pale-yellow liquid. The crude material was purified with silica gel column chromatography to yield ssPalmO-Paz4-C2 (1.1 g). 1H NMR (600 MHz, CDCl_3_): δ (ppm) = 0.86–0.90 (t, 6H), 1.25–1.34 (m, 40H), 1.58–1.64 (m, 4H), 1.99–2.03 (m, 8H), 2.28–2.32 (m, 4H), 2.45–2.70 (m, 24H), 2.80–2.85 (m, 4H), 4.18–4.21 (t, 4H), 5.13–5.38 (m, 4H).

### 2.5 Synthesis of ssPalmL

Compound **2** (0.80 g, 2.7 mmol) and Linoleic acid (1.7 g, 5.9 mmol) were dissolved in chloroform (8.1 mL) at 20-25 °C. Thereafter, 4-dimethylamino pyridine (0.13 g, 1.1 mmol) and 1-ethyl-3-(3-dimethylaminopropyl) carbodiimide hydrochloride (1.6 g, 8.1 mmol) were added to the solution. The mixture was then stirred at 30 °C for 3 hours. The resulting mixture was evaporated to give a pale brown liquid. The concentrate was dissolved in hexane (13 mL), and acetonitrile (6.2 mL) was added to the solution. The hexane layer was recovered, and evaporated to give the crude ssPalmL as pale-yellow liquid. The crude material was purified with silica gel column chromatography to yield ssPalmL (0.83 g). 1H NMR (600 MHz, CDCl_3_): δ (ppm) = 0.87–0.91 (t, 6H), 1.25–1.38 (m, 28H), 1.60–1.68 (m, 4H), 1.78–1.81 (m, 4H), 2.03–2.07 (m, 8H), 2.23–2.31 (m, 10H), 2.44–2.47 (m, 4H), 2.66–2.70 (m, 4H), 2.75–2.82 (m, 8H), 4.10–4.13 (t, 4H), 5.32–5.40 (m, 8H).

### 2.6 Animal experiments

The experimental protocols were reviewed and approved by the Hokkaido University Animal Care Committee and Chiba University Animal Care Committee in accordance with the “Guide for the Care and Use of Laboratory Animals”.

### 2.7 Preparation of the dextran sulfate sodium (DSS)-induced colitis mice model

C57BL/6J mice (male, 6 weeks, 20–22 g) were purchased from Japan SLC (Shizuoka, Japan). The animals were used after an initial 1 week period of adaptation. After adaption period, the drinking water of the mice was replaced to a 2% solution of DSS in distilled water. The DSS solution was administered ad libitum to C57BL/6 mice and other breeding conditions were not changed [7]. The mice were weighed daily and visually inspected for rectal bleeding and diarrhea. Generally, the symptoms of colitis are observed from 5 days after administration.

### 2.8 Preparation of empty LNPs

All empty LNPs (for biocompatibility experiments) were prepared using an ethanol dilution method. The lipids composed of ssPalm/DOPC/Chol (30/40/30 in molar ratio, 2.5 μmol) plus 15 mol% (0.375 μmol) of PEG_2000_-DSG were dissolved in 400 μL of EtOH. As a ssPalm, ssPalmM, ssPalmO, or ssPalmL was used. In a second tube, 400 μL of a 20 mM malic acid buffer (pH 4.0) was prepared. These lipids and buffers were warmed at 37 °C. The lipid solution was then added to the buffer solution under conditions of vortexing. The mixed solution (EtOH: water = 1: 1) was then incubated at 37 °C for 30 min. After incubation, fourteen mL of phosphate buffered saline (PBS) was added. The diluted solution was concentrated by ultrafiltration using Amicon Ultra 15 (NMWL:100K, Merck Millipore, Germany) or Vivaspin Turbo 15 (NMWL:100K, Sartorius, Germany) at 1000 g for 20 min at 25 °C. The LNP solutions remaining on the upper column were again diluted with 14 mL of PBS, then concentrated again at 1000 g for 20 min at 25 °C. Finally, a volume of the LNP solution was adjusted to 1 mL with PBS. The diameter and ζ-potential of the LNPs were determined using a dynamic light scattering spectrophotometer (Zetasizer nano; Malvern Instruments Ltd., Malvern, WR, UK). Measurement temperature was adjusted to 25 °C.

### 2.9 Evaluation of biocompatibility of LNP_ssPalm_ with a series of hydrophobic scaffolds

DSS-colitis model mice were intravenously injected with 200 μL of the empty LNP solution (10 mM total lipids concentration) from Day 1 to Day 5 during colitis induction. As an index of colitis, the weights of the mice were measured daily after administration of DSS solution. At day 9, the mice were sacrificed and dissected. Colon lengths were measured from the bottom of the cecum to the aboral end of the rectum.

### 2.10 In vitro transcription (IVT) of mRNA

pDNA containing Luciferase cassette under a T7 promoter (pcDNA3.1-(+)-Luc(0)) was used as a template for the in vitro transcription. The pDNA was linearized using EcoRV-HF (New England Biolab). One μg of the linear DNA was transcribed and polyadenylated using a mMESSAGE mMACHINE T7 Ultra Kit (Thermo Fisher Scientific) according to the manufacturer's protocol.

### 2.11 Preparation of the mRNA encapsulating LNP_ssPalm_

mRNA solutions were prepared in malic acid/NaOH buffer (pH3.0, 20 mM malic acid and 30 mM NaCl) at a concentration of 0.067 μg/μL (30 μg mRNA/450 μL malic acid buffer). The lipid mixture was prepared in 300 μL of EtOH. The composition of lipids was ssPalm (O or O-Paz4-C2)/DOPC/cholesterol = 60/10/30 (1315 nmol in total) with additional PEG_5000_-DSG (1.5–8 mol%). The mRNA solution was rapidly mixed with the ethanol solution of the lipid at room temperature under vortexing. Then, 10 mL of MES/NaOH buffer (pH5.5, 20 mM) was added to the mixture. The LNP_ssPalm_ solution was concentrated by ultrafiltration using an Amicon Ultra 15 (NMWL:100K) or Vivaspin Turbo 15 (NMWL:100K) at 1000 g for 20 min at 25 °C. The concentrated LNP_ssPalm_ solutions remaining on the upper column were again diluted with 14 mL of PBS (buffer exchange), then concentrated again at 1000 g for 20 min at 25 °C.

### 2.12 In vivo luciferase assay

DSS-colitis mice at day 5 were administered with the LNP_ssPalm_ (20 μg mRNA/mouse). After 4 hours, mice were sacrificed and organs (colon, liver, spleen, kidney, lungs, and heart) were dissected. Fifty mg of each organ was collected in micro tubes and rapidly frozen in liquid nitrogen. The samples were stored at −80 °C until measurements. The samples were homogenized in 800 μL of *in vivo* lysis buffer (pH7.4, 100 mM Tris/HCl, 2 mM EDTA, 0.1 w/v% Triton X-100) using Micro Smash MS-100R (TOMY SEIKO, Tokyo, Japan) at 3000 rpm for 1 min. The homogenization was repeated twice. Then, the tubes were centrifuged (10 min, 4 °C, 13000 rpm) and supernatants were collected. The luciferase activity and protein concentration of the supernatant was measured using a Luciferase Assay System (Promega) and BCA Protein Assays (Thermo Fisher Scientific), respectively.

## 3 Results and discussion

An increasing number of reports indicated the existence of a relationship between the structure of hydrophobic scaffolds of delivery systems and their toxicity by using healthy mice [8]. However, in a pathological state such as an inflammatory disease, the effects of hydrophobic scaffolds have not been investigated. We thus analyzed the effects of the structure of hydrophobic scaffold in a ssPalm molecule on inflammatory reactions by using the DSS-colitis model. The scaffolds of ssPalm used in this study were myristic acid (ssPalmM), oleic acid (ssPalmO), and linoleic acid (ssPalmL). Chemical structures of these ssPalm materials were shown on Fig. 1a. The physicochemical properties of the empty LNPs without nucleic acids, prepared with these ssPalms (LNP_ssPalmM_, LNP_ssPalmO_, and LNP_ssPalmL_) were summarized in Fig. 1b and Table 1. Since the size of these nanoparticles was approximately 110 nm, the particles were nearly optimal size for the accumulation to the inflammatory lesions in colon as we reported previously [4].

**Table 1:** Properties of LNP_ssPalms_.

Lipids (ssPalm)	Particle size (nm)	PdI	Zeta potential (mV)
ssPalmM	117 ± 8	0.11 ± 0.02	3.3 ± 1.0
ssPalmO	93 ± 11	0.08 ± 0.01	1.5 ± 0.5
ssPalmL	140 ± 8	0.09 ± 0.02	2.0 ± 0.8

Particle size, PdI, and Zeta potential were measured using dynamic light scattering.

**Fig. 2:**
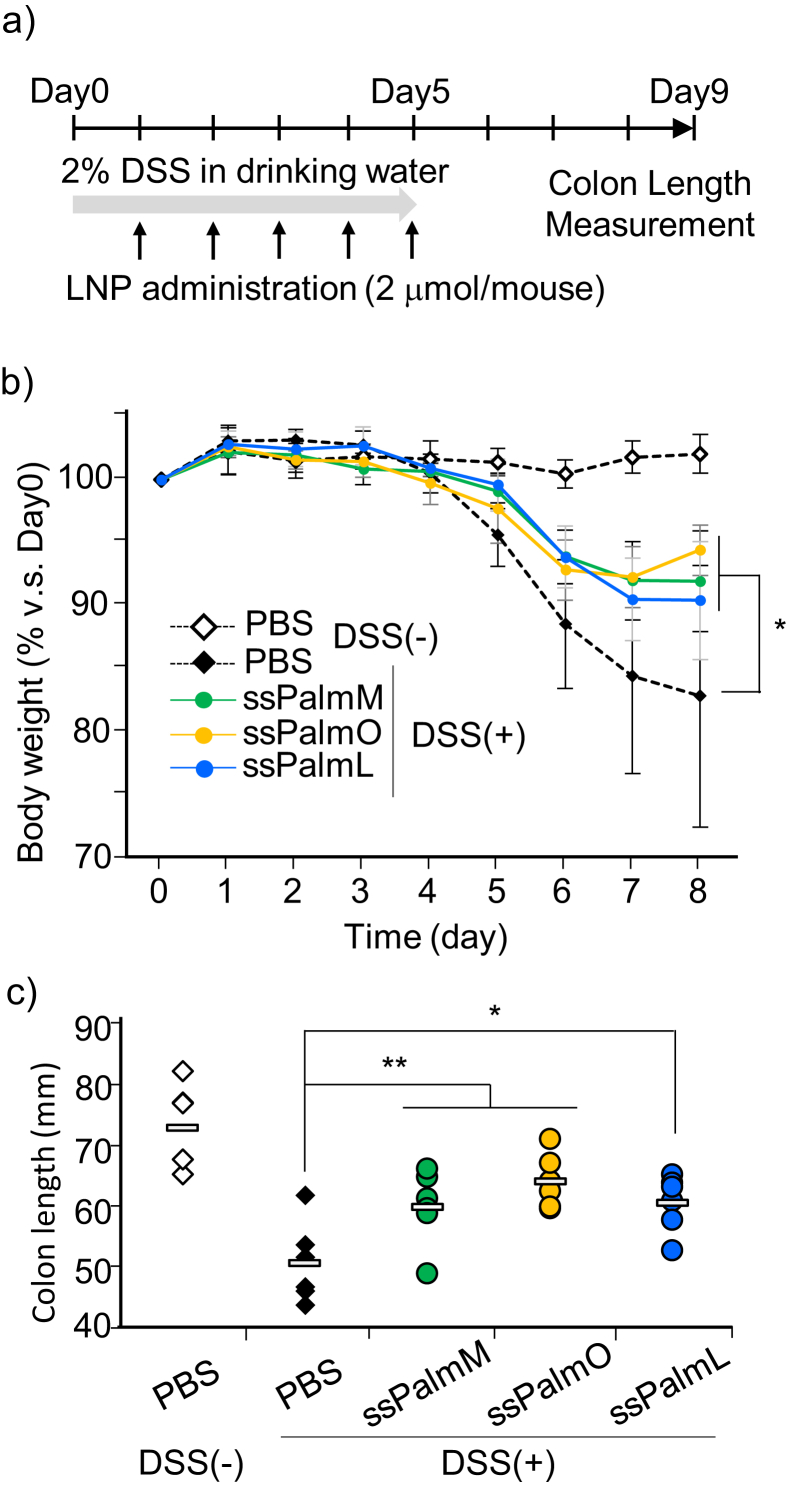
a) Experimental design for evaluating biocompatibility. DSS-colitis was established by administrating 2% DSS in drinking water for 5 days. The empty LNP_ssPalm_ was administered intravenously at a dose of 2 μmol lipid/mouse. Body weight and colon length were measured as an index of inflammation in colon. b) Changes in body weight. The body weight from day 1 to day 8 was normalized by those of day 0. The data are represented as the mean ± SD (n = 6). c) Evaluation of colon length. At day 9, mice were sacrificed, and colon samples were dissected. The colon lengths were measured from the bottom of the cecum to the aboral end of the rectum. The data are represented as the average (rectangle) with individual values (diamonds and circles) (n = 6). Statistical analyses were performed by One-way ANOVA followed by Dunnett's test (*; p < 0.05, **; p < 0.01).

The DSS-colitis was established by administrating 2% DSS in drinking water for 5 days (Fig. 2a). The empty-LNP_ssPalm_ was administered intravenously at a dose of 2 μmol lipid/mouse for 5 days. This dose is corresponding to approximately 70 mg/kg total lipids. As a result, the daily intravenous administration of high dose of LNP_ssPalm_ significantly alleviated the weight loss of the colitis model regardless of the structure of hydrophobic scaffolds (Fig. 2b). The alleviation of the colitis was further confirmed by measuring the colon length at day 9. The reduction in colon length was also significantly ameliorated by the administration of all three LNP_ssPalms_ compared to the PBS group (Fig. 2c).

It has been reported that an inhibition of endosomal maturation by neutralizing the compartment resulted in the attenuation of inflammatory signaling pathway, which is triggered by a certain kinds of pattern recognition receptors (i.e. Toll-like receptors). This mode of immune suppression was applied for the treatment for DSS-colitis; the neutralization of the endosomal pH by artificial proton-sponging peptides successfully alleviated the aggravation of the colitis [9]. Thus, it is likely that the alleviating effects of the LNP_ssPalms_ on the DSS-colitis model is partially due to the proton-accepting property of tertiary amine units of the ssPalm. Among the LNP_ssPalms_, the LNP_ssPalmO_ tended to alleviate the inflammation more efficiently than the LNP_ssPalmM_ and LNP_ssPalmL_. This observation can be explained by the innate biological functions of the fatty acid scaffolds. It is known that saturated fatty acids, including myristic acid, function as ligands for TLR-4. While lauric acid and palmitic acid are mostly known for their immune stimulation activity, myristic acid has also been reported to be involved in the exacerbation of hepatitis [10]. In addition, linoleic acid, an omega-6 fatty acid, can be metabolized to other omega-6 fatty acids such as arachidonic acid. The arachidonic acid is eventually converted to prostaglandins, which mediate inflammation [11]. On the other hand, oleic acid is an omega-9 fatty acid, a class of compounds that are known to be relatively immune-suppressive. It was recently reported that an oleic acid treatment decreased the production of inflammatory cytokines and the cellular proliferation of a line of T cell derived cells [12]. Thus, the oleic acid is one of the most potent hydrophobic scaffolds for ionizable lipids from the point of view of biocompatibility, especially with respect to delivery to the inflammatory lesions.

**Fig. 3:**
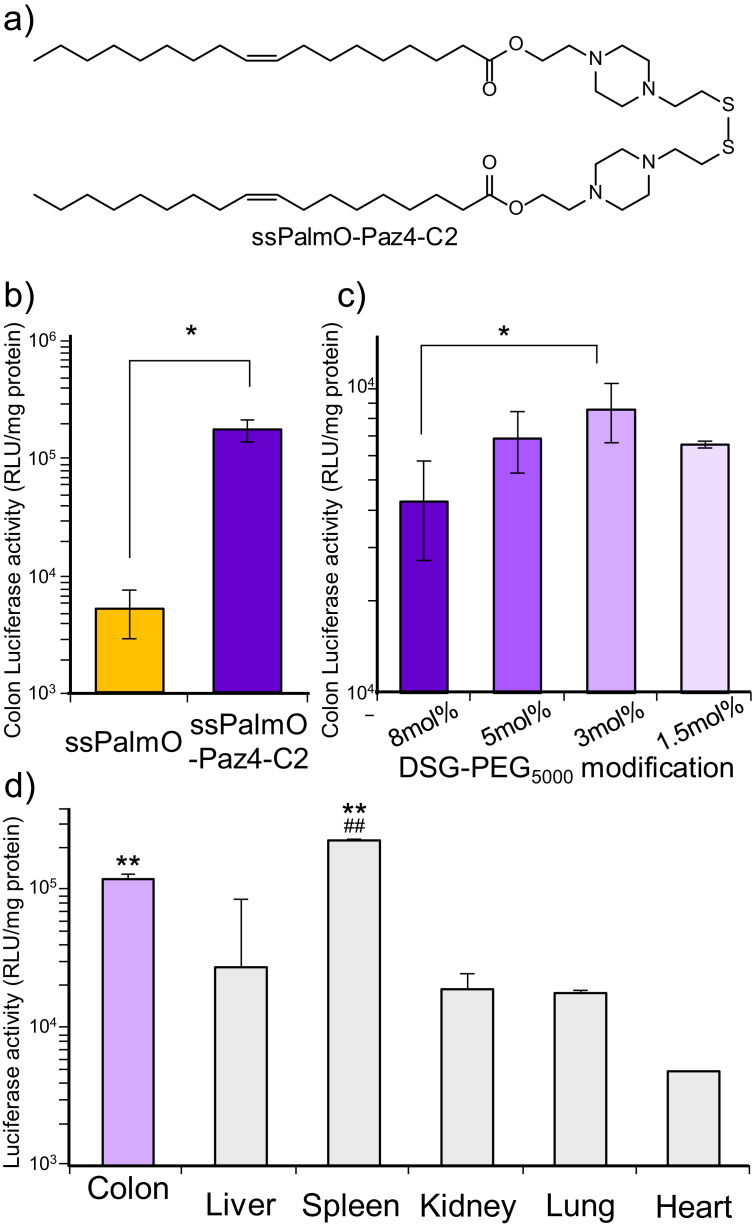
a) Chemical structure of the ssPalmO-Paz4-C2. The synthesis of the ssPalmO-Paz4-C2 was described in Materials and methods section. b) Transgene activity of the LNP_ssPalm_ to the colon of DSS-colitis mouse (day 5). LNP_ssPalm_ containing luciferase-mRNA was administrated to the DSS-colitis mouse. Colon was dissected 4 hours after administration. Statistical analysis was performed by means of the student's t-test (*; p < 0.05). c) Transgene activity of LNP_ssPalm_ with different surface PEGylation (1.5–8 mol% of total lipids) to the colon of DSS-colitis mouse (day 5). Statistical analyses were performed by One-way ANOVA followed by SNK test (*; p < 0.05). d) Organ distribution of the luciferase expression. Four hours after administration, organs (Colon, Liver, Spleen, Kidney, Lung, and Heart) were dissected and luciferase activity was determined. Statistical analyses were performed by One-way ANOVA followed by SNK test (**; p < 0.01 v.s. Liver, Kidney, Lung, and Heart, ^##^; p < 0.01 v.s. Colon). Each bar indicated mean ± SD (n = 3).

Next, the tertiary amine groups of the ssPalmO was modified to increase endosomal escape efficiency of the mRNA. For stabilizing the protonated state of the tertiary amine groups, the tertiary amine was fixed into the piperazine ring of the headgroups (ssPalmO-Paz4-C2, Fig. 3a) [13]. The structural modification from flexible amines of the original ssPalm (ssPalmO) to the piperazine ring (ssPalmO-Paz4-C2) resulted in the enhancement of the transgene expression by over 10-fold in the inflammatory colon of DSS-colitis model (Fig. 3b). We next optimized the effect of the surface modification of the particle with polyethylene glycol (PEG_5000_-DSG) on the transgene activity of the LNP_ssPalmO-Paz4-C2_ (Fig. 3c). In the case of neutral LNPs, surface modification with a hydrophilic polymer is necessary to prevent aggregation. However, it is generally observed that LNPs modified with PEG-lipids containing shorter fatty acid scaffolds (i.e. dimyristoyl scaffold; PEG-DMG) dominantly accumulate in the liver, when the particles are administrated via the tail vein since the shorter PEG-lipids can easily dissociate from the particles in the blood circulation [6, 14, 15, 16]. Since a longer blood retention is a crucial driving force for delivering LNP_ssPalm_ to an inflammatory lesion, the LNP_ssPalms_ used in the present study were modified with PEG-lipids that contain a longer chain fatty acid (distearoyl scaffold; PEG-DSG) that would allow the particles to be retained for longer periods of time in the blood. However, the use of PEG-DSG is attended by a dilemma: PEG modification has merit in avoiding hepatic clearance and prolonging the blood circulation time of the particles, while it, in parallel, prevents the cellular uptake and endosomal escape after the cellular entry via endocytosis. Thus, the density of the PEG and the type of PEG-lipid need to be optimized so as to achieve to maximize and minimize the mRNA transfection activity in the target organ (colon) and non-target organ (liver), respectively [17, 18]. The LNP_ssPalms_ were modified with PEG_5000_-DSG at levels from 1.5 to 8.0 mol% of the total lipid and transgene expression in the colon was determined. The physicochemical properties of these PEG-modified particles are shown in Table 2. As a result, a 3 mol% modification with PEG_5000_-DSG was found to be suitable for mRNA expression in the inflammatory colon. In the case of the LNP_ssPalms_ used in this study, the amount of the PEG-lipids grafted onto the particles had no effect on the particle size, PdI or Zeta potential. The expression of luciferase was mainly detected in colon and spleen (Fig. 3d). It is noteworthy that the LNP_ssPalmO-Paz4-C2_ exhibited a higher transgene efficiency in the colon compared to the liver, which suggests that 3 mol% modification with PEG_5000_-DSG was suitable for balancing the efficient colon targeting of the particles and the cytoplasmic delivery of the mRNA.

**Table 2:** Properties of LNP_ssPalms_ modified with different amount of DSG-PEG.

Amount of PEG	Particle size (nm)	PdI	Zeta potential (mV)
1.5% DSG-PEG	117 ± 7	0.11 ± 0.01	−3.6 ± 0.6
3% DSG-PEG	117 ± 1	0.09 ± 0.01	−1.5 ± 0.3
5% DSG-PEG	114 ± 5	0.11 ± 0.02	−0.4 ± 0.2
8% DSG-PEG	119 ± 2	0.12 ± 0.02	−0.3 ± 0.9

The composition of the particles was ssPalmO-Paz4-C2/DOPC/Chol = 6/1/3. The indicated mol% of DSG-PEGs were additionally added in lipid composition.

In conclusion, oleic acid was found to be one of the most suitable hydrophobic scaffolds for the delivery of mRNA to inflammatory lesion. The presence of the piperazine rings in the headgroup of the ionizable lipid caused a significantly higher transgene activity compared to the flexible tertiary amines of the original ssPalm. Collectively, the LNP_ssPalm_ that was composed of ssPalmO-Paz4-C2 appears to a promising mRNA vector for realizing gene therapy for inflammatory diseases such as the ulcerative colitis.

## Declarations

### Author contribution statement

Hiroki Tanaka: Conceived and designed the experiments; Performed the experiments; Analyzed and interpreted the data; Wrote the paper.

Ayaka Watanabe, Manami Konishi: Performed the experiments.

Yuta Nakai, Hiroki Yoshioka, Tatsuya Ohkawara, Hiroshi Takeda: Analyzed and interpreted the data; Contributed reagents, materials, analysis tools or data.

Hideyoshi Harashima: Conceived and designed the experiments.

Hidetaka Akita: Conceived and designed the experiments; Wrote the paper.

### Funding statement

This work was supported by the JSPS KAKENHI [grant numbers 17H06558, 17K19473, 18K18377] and the JST CREST [grant number JPMJCR17H1]. HA is also supported by the Asahi Glass Foundation.

### Competing interest statement

The authors declare the following conflicts of interest: Hokkaido University and the NOF CORPORATION hold patent-pending (WO2013/073480 and WO2016/121942) on the ssPalm chemicals. H.T., Y.N., H.H., and H.A. are the inventors of the patent.

### Additional information

No additional information is available for this paper.
